# Regulators of Collagen Fibrillogenesis during Molar Development in the Mouse

**DOI:** 10.3389/fphys.2017.00554

**Published:** 2017-08-02

**Authors:** Ivana Zvackova, Eva Matalova, Herve Lesot

**Affiliations:** ^1^Institute of Animal Physiology and Genetics, Academy of Sciences of the Czech Republic Brno, Czechia; ^2^Department of Physiology, University of Veterinary and Pharmaceutical Sciences Brno, Czechia; ^3^Biology Department, Ghent University Ghent, Belgium

**Keywords:** odontogenesis, alveolar bone, FACITs, SLRPs, collagen

## Abstract

Development of mammalian teeth and surrounding tissues includes time–space changes in the extracellular matrix composition and organization. This requires complex control mechanisms to regulate its synthesis and remodeling. Fibril-associated collagens with interrupted triple helices (FACITs) and a group of small leucine-rich proteoglycans (SLRPs) are involved in the regulation of collagen fibrillogenesis. Recently, collagen type XII and collagen type XIV, members of the FACITs family, were found in the peridental mesenchyme contributing to alveolar bone formation. This study was designed to follow temporospatial expression of collagen types XIIa and XIVa in mouse first molar and adjacent tissues from embryonic day 13, when the alveolar bone becomes morphologically apparent around the molar tooth bud, until postnatal day 22, as the posteruption stage. The patterns of decorin, biglycan, and fibromodulin, all members of the SLRPs family and interacting with collagens XIIa and XIVa, were investigated simultaneously. The situation in the tooth was related to what happens in the alveolar bone, and both were compared to the periodontal ligament. The investigation provided a complex localization of the five antigens in soft tissues, the dental pulp, and periodontal ligaments; in the mineralized tissues, predentin/dentin and alveolar bone; and junction between soft and hard tissues. The results illustrated developmentally regulated and tissue-specific changes in the balance of the two FACITs and three SLRPs.

## Introduction

Mammalian tooth and jaw-bone development has mainly been investigated in the lower jaw of the mouse (Peterkova et al., 2000; Chlastakova et al., 2011; Lungova et al., 2011; Gama et al., 2015). The steps of morphogenesis, histogenesis, and cell differentiation are largely integrated. They are regulated by cell interactions as well as by short- and long-range molecular signaling (Peters and Balling, 1999; Miletich et al., 2011; Jussila and Thesleff, 2012). The extracellular matrix (ECM) plays an important role and, for this reason, has been investigated by means of structural and functional analyses (MacDonald and Hall, 2001; Kleinman et al., 2003; Fukumoto and Yamada, 2005; Rodriguez et al., 2010). This ECM consists of collagens, non-collagenous proteins, and proteoglycans. The composition of the ECM varies in space and time during development, meaning complex control mechanisms regulate its synthesis, organization, and remodeling (Kaku and Yamauchi, 2014; Takimoto et al., 2015; Xu et al., 2016).

Twenty-nine different collagens have been identified so far (Home-Gene-NCBI)^1^. Subfamilies have been distinguished, depending on their structural organization linked to supramolecular assemblies: fibril-forming collagens, fibril-associated collagens with interrupted triple helices (FACITs), network-forming collagens, membrane collagens, multiplexins, and, out of these groups, collagens VI, VII, XXVI, and XXVIII (for review, see Ricard-Blum, 2011). Molecules regulating collagen fibrillogenesis and spatial organization of fibers (Canty and Kadler, 2005; Chen et al., 2015) include FACITs (Ricard-Blum, 2011) and a group of small leucine-rich proteoglycans (SLRPs; Schaefer and Iozzo, 2008; Kalamajski and Oldberg, 2010; Chen and Birk, 2013).

FACITs are multi-domain proteins with several triple-helical regions separated by non-collagenous domains. FACITs, which exist as single molecules, include collagens IX, XII, XIV, XVI, XIX, XX, XXI, and XXII (Ricard-Blum, 2011). Two members of these FACITs were investigated in the present study, collagens XIIa and collagen XIVa, which were recently identified in mesenchymal cells contributing to alveolar bone formation (Minarikova et al., 2015).

Different isoforms of collagen XII (XIIa and XIIb) exist, which are generated by alternative splicing (Kania et al., 1999). Moreover, collagen XIIa, but not XIIb, can occur as a proteoglycan, with glycosaminoglycan side chain(s) attached to the alternatively spliced NC3 region (Chiquet et al., 2014). Collagens XII and XIV bind decorin (DCN) and biglycan (BGN), and both of them show significant structural homologies. Nevertheless, it has been suggested that collagen XII might indirectly bridge adjacent collagen fibers (Shaw and Olsen, 1991), whereas collagen XIV would interfere with fibril elongation (Young et al., 2000). The large non-collagenous domain (NC3) of collagen XIV projects into the interfibrillar space (Keene et al., 1991). This domain has been implicated in the initial regulation of fibril packing in the tendon (Chen et al., 2015), possibly by inhibiting lateral fibril growth (Ansorge et al., 2009).

SLRPs are encoded by 18 genes and divided into five classes (for review, see Iozzo and Schaefer, 2015). Several members of class I, such as DCN and BGN, and class II, such as fibromodulin (FMOD), regulate collagen fibrillogenesis, although in different ways (Douglas et al., 2006). SLRPs can interact with network-forming and fibrillar collagens as well as with FACITs, such as type XII and type XIV collagens (Font et al., 1993, 1998; Kalamajski and Oldberg, 2010). In this latter case, they can cooperate to regulate linear and lateral collagen fibril growth (Chen et al., 2015). Ansorge et al. (2009) suggest that time-controlled replacement of type XIV collagen by other fibril-associated molecules such as type XII collagen or SLRPs would allow continued regulation of collagen fibers' lateral growth. SLRPs can also influence mineralization (Chen et al., 2004; Goldberg et al., 2006; Haruyama et al., 2009; Young, 2016) and be involved in signaling (Schaefer and Iozzo, 2008; Gubbiotti et al., 2016).

The timing of protein expression and the localization of collagens XIIa and XIVa have been investigated in the periodontal ligament (PDL; Zhang et al., 1993; Tsuzuki et al., 2016), but neither in the developing tooth nor in the alveolar bone. For this reason, a specific study was designed covering prenatal as well as postnatal stages in mouse first lower molar and periodontium from embryonic day 13 (E13), when the forming alveolar bone becomes morphologically apparent around the molar tooth bud, until postnatal day 22 (P22), as the posteruption stage, when the tooth is dynamically anchored. The patterns of DCN, BGN, and FMOD, which all interact with collagens XIIa and XIVa, were investigated simultaneously. The situation in the tooth was compared to what happens in the alveolar bone, and both were compared to the PDL used as a reference tissue.

The major aim of this investigation was to search for developmentally regulated and tissue-specific changes in the balance of different regulators of collagen fibrillogenesis.

## Materials and methods

### Animals

Wild- type mice (strain CD-1) were purchased from the Breeding Units of Masaryk University Brno. Pregnant mice were euthanized according to the experimental protocol, approved by the Laboratory Animal Science Committee of the IAPG CAS, v.v.i., Czech Republic. Day 0 of pregnancy was determined as the mating day (controlled mating for 2 h). Heads of pups corresponding to embryonal (E) days 13, 14, 15, 18 and postnatal (P) days 0, 11, 22 were sampled. Heads or dissected quadrants of mandibles were fixed in 4% buffered paraformaldehyde, fully decalcified in phosphate-buffered 0.5 M EDTA (Sigma Aldrich, St. Louis, MO), dehydrated in ethanol series, treated with xylene and embedded in paraffin. Serial frontal histological sections were processed and split between slides for the following analyses: haematoxylin-eosin (HE) for morphology staining; and immunohistochemistry. For the best illustrations, the same sequence of serial frontal sections was always maintained as: histology; biglycan (BGN); collagenXIIa (coll12a); collagenXIVa (coll14a); fibromodulin (FMOD); and decorin (DCN).

### Immunohistochemistry

The samples were deparaffinized (xylen), rehydrated (a gradient series of ethanol) and the antigen retrieval was applied for collagen XIVa (citrate buffer, pH = 6.0/98°C/10 min/water bath). Endogenous peroxidase activity was eliminated in all sections by 3% hydrogen peroxidase in phosphate- buffered saline (collagen XIVa RT/1 min, collagen XIIa, fibromodulin, biglycan and decorin RT/10 min) to eliminate possible background. The primary antibodies Anti-collagen XIV α1 1:50 (Invitrogen PA5-49916); Anti-collagen XII α1 1:100 (Sigma-Aldrich US/SAB4500395) were applied overnight at 4°C. The primary antibodies rabbit polyclonal Anti- biglycan 1:800 (LF-159), Anti-decorin 1:800 (LF-114), Anti-fibromodulin 1:800 (LF-150) (LF-kind gifts from Dr. Larry Fisher, NIDCR/NIH, Bethesda, MD) were applied for 2 h at room temperature. To visualize the primary antibody, a peroxidase- conjugated streptavidin- biotin system (Vectasin PK-4002; Vector Laboratories, Inc. Burlingame, CA) followed by chromogen substrate diaminobenzidine (DAB; K3466; Dako, Copenhagen, Denmark) reaction were applied. Cells were counterstained with haematoxylin to visualize the nuclei (blue). Negative controls were performed by omitting the primary antibody from the reaction mixture (Supplementary Figure 1).

## Results

**Figure 1:**
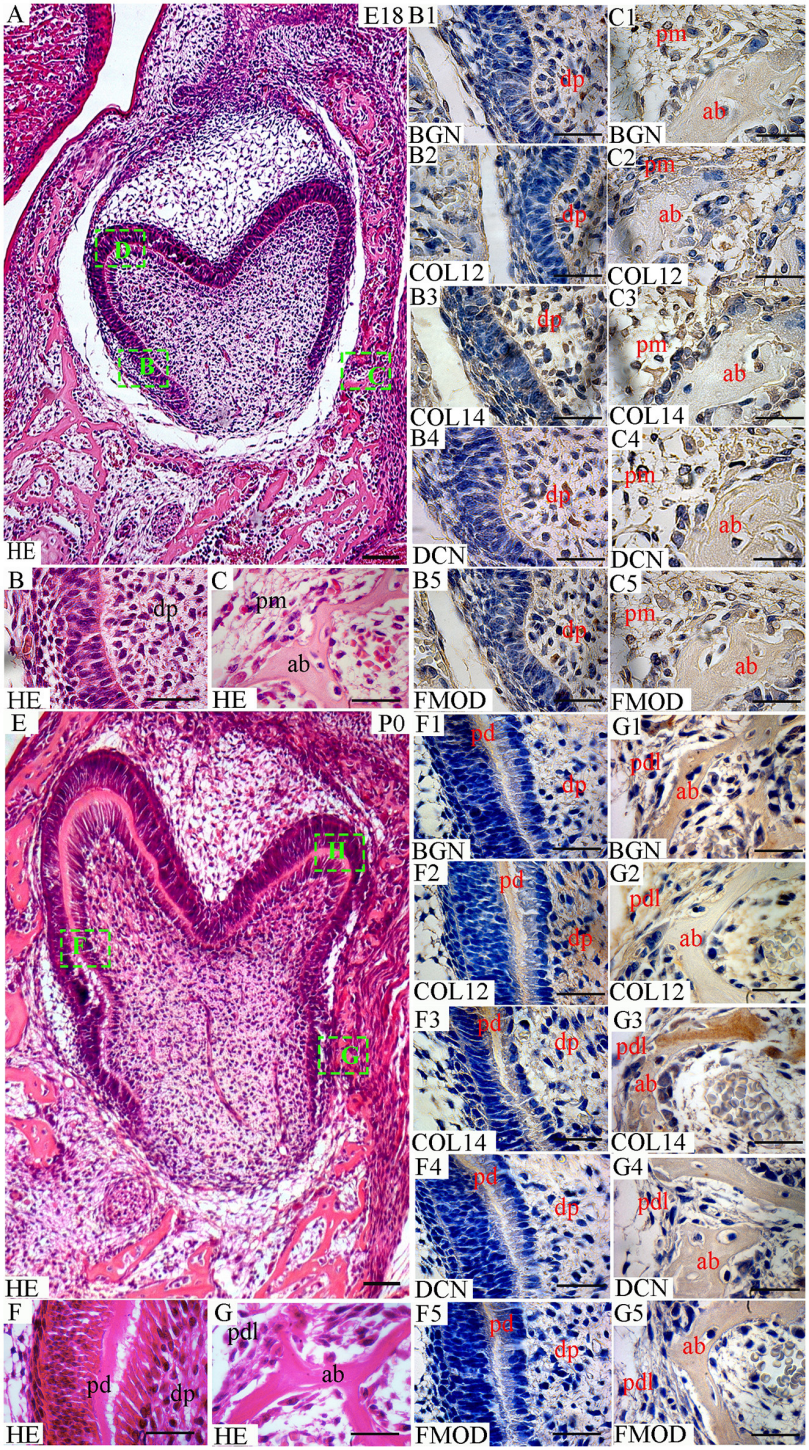
Localization of FACITs and SLRPs in the developing tooth and surrounding structures at embryonic day 18 (E18) and perinatal day (P0). **(A)** Haematoxylin and eosin staining of embryonic tooth and surrounding tissues (E18). Details from boxes (B–D) in **A** is shown in **B,C**, and Figure 2D. **(B)** Detail of dental pulp. **(C)** Detail of peridental mesenchyme and alveolar bone. Expression of biglycan in dental **(B1)** and peridental mesenchymes **(C1)**. Expression of collagen XIIa in dental **(B2)** and peridental mesenchymes **(C2)**. Expression of collagen XIVa in dental **(B3)** and peridental mesenchymes **(C3)**. Expression of decorin in dental **(B4)** and peridental mesenchymes **(C4)**. Expression of fibromodulin in dental **(B5)** and peridental mesenchymes **(C5)**. **(E)** Haematoxylin and eosin staining of the tooth and surrounding tissues at P0. Details from boxes (F–H) in **E** is shown in **F**,**G**, and Figure 2H. **(F)** Detail of dental pulp and predentin. **(G)** Detail of periodontal ligaments and alveolar bone. Expression of biglycan **(F1)**, collagen XIIa **(F2)**, collagen XIVa **(F3)**, decorin **(F4)**, and fibromodulin **(F5)** in predentin and dental pulp. Expression of biglycan **(G1)**, collagen XIIa **(G2)**, collagen XIVa **(G3)**, decorin **(G4)**, and fibromodulin **(G5)** in the periodontal ligament and alveolar bone. Dp, dental pulp; pm, peridental mesenchyme; ab, alveolar bone; pd, predentin; pdl, periodontal ligaments; BGN, biglycan; COL12, collagen type XIIa; COL14, collagen type XIVa; DCN, decorin; FMOD; fibromodulin; HE, haematoxylin/eosin. Scale bar **(A,E)** = 100 μm. Scale bar **(B,B1–B5,C,C1–C5,F,F1–F5,G,G1–G5)** = 25 μm.

**Figure 2:**
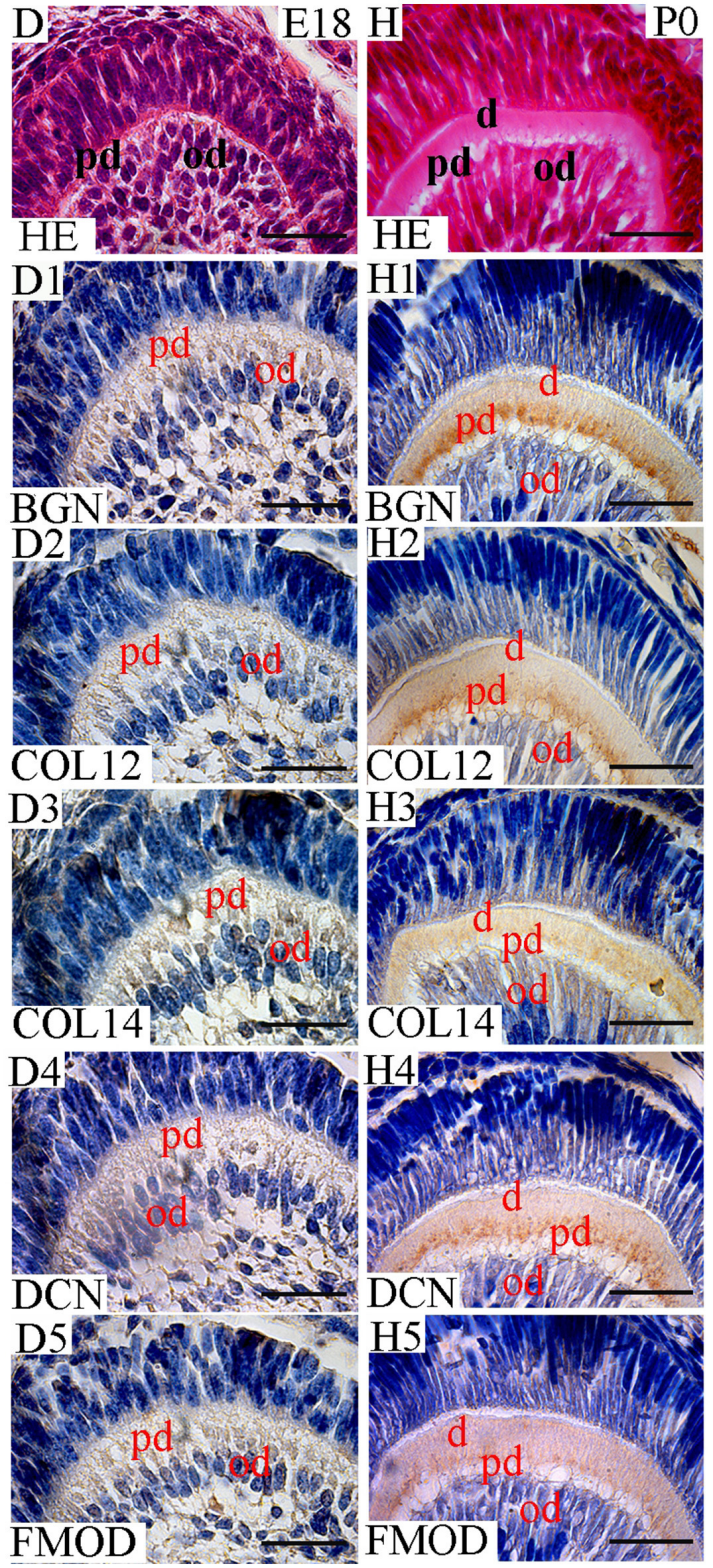
Localization of FACITs and SLRPs in predentin and dentin at E18 and P0. **(D)** Detail of dental pulp at E18 and **(H)** P0. Expression of biglycan (**D1**), collagen XIIa **(D2)**, collagen XIVa **(D3)**, decorin **(D4)**, and fibromodulin **(D5)** in the dental pulp cells and odontoblast layer at E18. Expression of biglycan **(H1)**, collagen XIIa **(H2)**, collagen XIV **(H3)**, decorin **(H4)**, and fibromodulin **(H5)** in predentin and dentin at P0. Pd, Predentin; od, odontoblast; d, dentin; BGN, biglycan; COL12, collagen type XIIa; COL14, collagen type XIVa; DCN, decorin; FMOD; fibromodulin; HE, haematoxylin/eosin. Scale bar = 25 μm.

**Figure 3:**
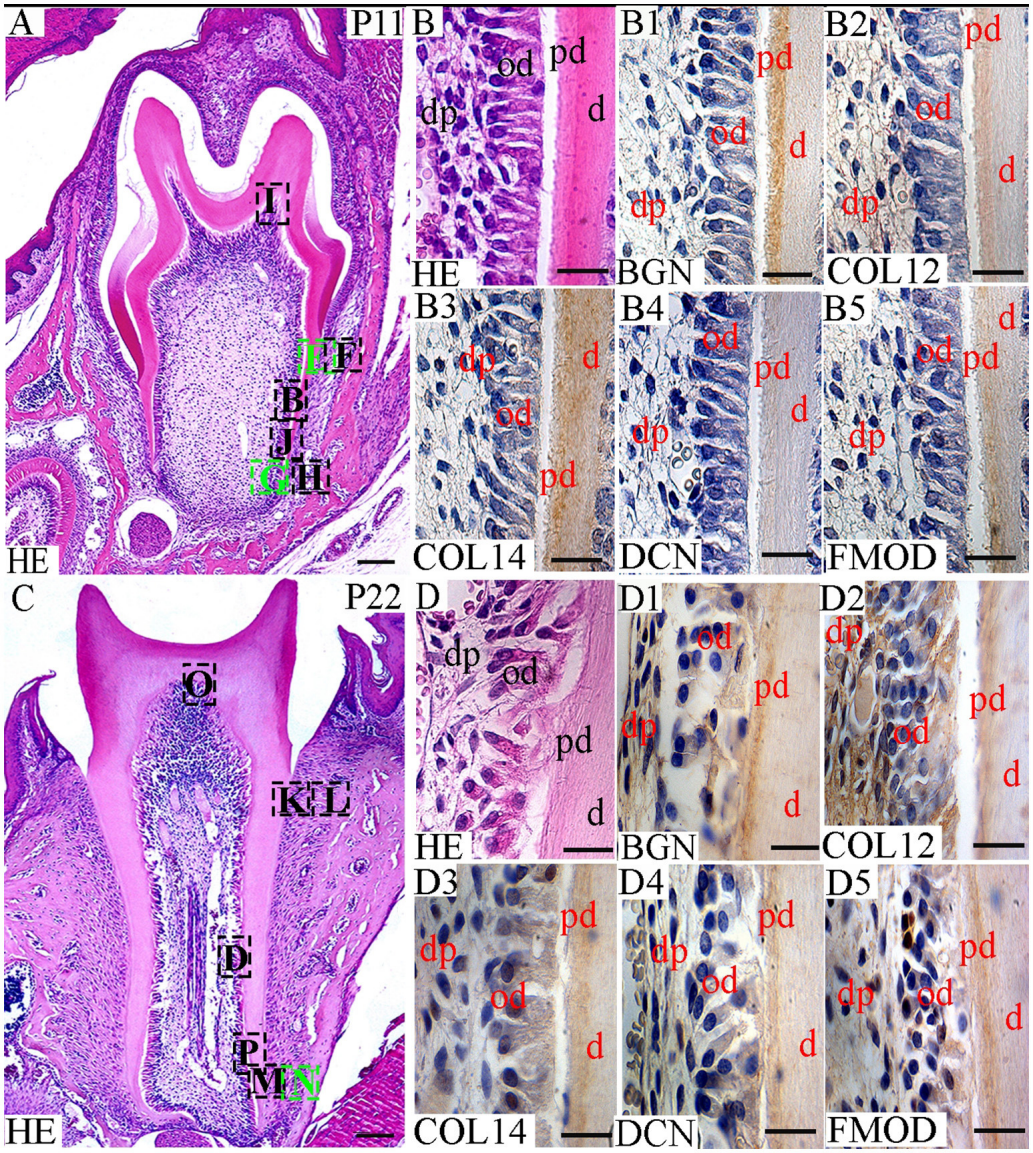
Localization of FACITs and SLRPs in dental pulp, predentin and dentin at P11 and P22. **(A)** Haematoxylin and eosin staining of the tooth and surrounding tissues at P11. Details from box B is shown in **B**, from boxes E–H are shown in Figures 4E–H, from boxes I and J in Figures 6I,J. **(C)** Haematoxylin and eosin staining of the tooth and surrounding tissues at P22. Details from box D is shown in **D**, from boxes K, L, M, N in Figures 5K–N, and from boxes O and P in Figures 6O,P. Detail of the pulp, predentin, and dentin at P11 **(B)** and P22 **(D)**. Expression of biglycan **(B1)**, collagen XIIa **(B2)**, collagen XIVa **(B3)**, decorin **(B4)**, and fibromodulin **(B5)** in the dental pulp, predentin and dentin at P11. Expression of biglycan **(D1)**, collagen XIIa **(D2)**, collagen XIVa **(D3)**, decorin **(D4)**, and fibromodulin **(D5)** in the dental pulp, predentin and dentin at P22. Od, Odontoblast; pd, Predentin; d, dentin; BGN, biglycan; COL12, collagen type XIIa; COL14, collagen type XIVa; DCN, decorin; FMOD; fibromodulin; HE, haematoxylin/eosin. Scale bar = 25 μm.

**Figure 4:**
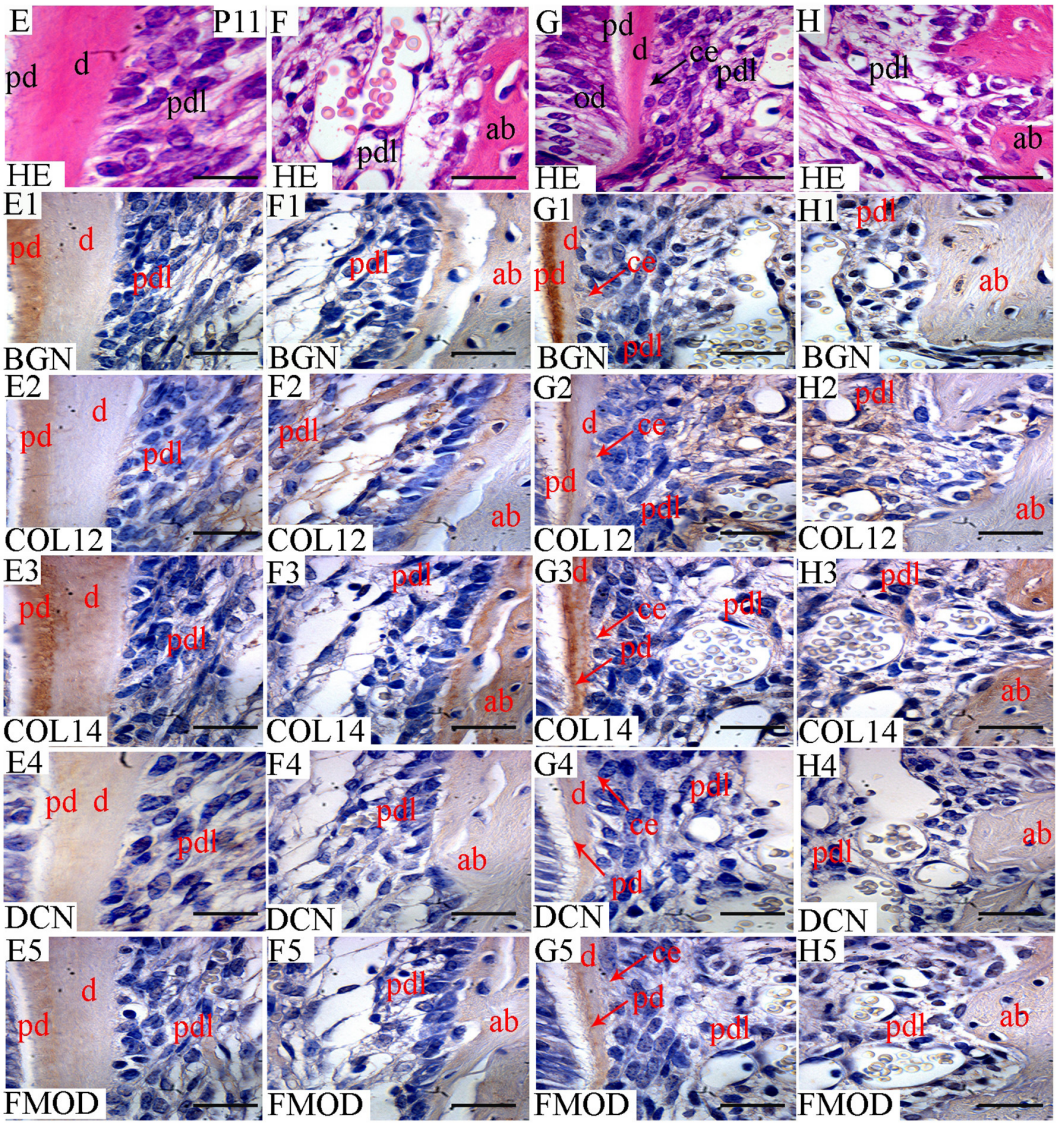
Localization of FACITs and SLRPs in predentin, dentin, periodontal ligaments, and alveolar bone at P11. Haematoxylin eosin staining of predentin/dentin and PDL in the crown **(E)** and root portions **(G)**. Haematoxylin eosin staining of PDL and alveolar bone in the crown portion **(F)** and root portion **(H)**. Expression of biglycan in predentin/dentin **(E1)**, PDL **(E1,F1)** and alveolar bone **(F1)** in the crown portion; predentin/dentin/cementum **(G1)**, PDL **(G1,H1)** and alveolar bone **(H1)** in the root portion. Expression of collagen XIIa in predentin/dentin **(E2)**, PDL **(E2,F2)** and alveolar bone **(F2)** in the crown portion; predentin/dentin/cementum **(G2)**, PDL **(G2,H2)** and alveolar bone **(H2)** in the root portion. Expression of collagen XIVa in predentin/dentin **(E3)**, PDL **(E3,F3)** and alveolar bone **(F3)** in the crown portion; predentin/dentin/cementum **(G3)**, PDL **(G3,H3)** and alveolar bone **(H3)** in the root portion. Expression of decorin in predentin/dentin **(E4)**, PDL **(E4,F4)** and alveolar bone **(F4)** in the crown portion; predentin/dentin/cementum **(G4)**, PDL **(G4,H4)** and alveolar bone **(H4)** in the root portion. Expression of fibromodulin in predentin/dentin **(E5)**, PDL **(E5,F5)** and alveolar bone **(F5)** in the crown portion; predentin/dentin/cementum **(G5)**, PDL **(G5,H5)** and alveolar bone **(H5)** in the root portion. Pd, Predentin; d, dentin; pdl, Periodontal ligaments; ab, alveolar bone; ce, cementum; BGN, biglycan; COL12, collagen type XIIa; COL14, collagen type XIVa; DCN, decorin; FMOD; fibromodulin; HE, haematoxylin/eosin. Scale bar = 25 μm.

**Figure 5:**
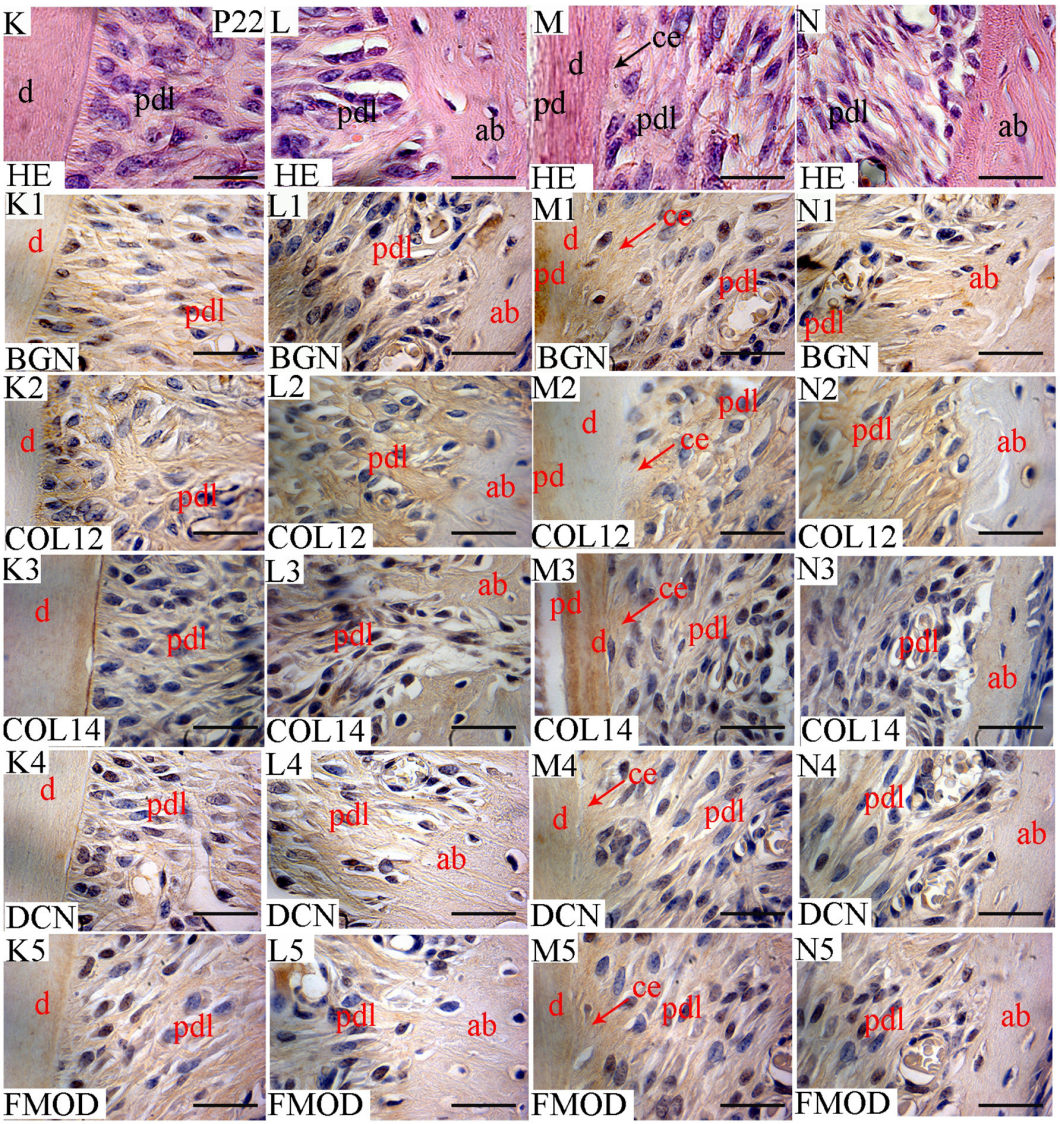
Localization of FACITs and SLRPs in predentin/dentin, periodontal ligaments and alveolar bone at P22. Haematoxylin eosin staining of dentin and PDL in the crown area **(K)** and predentin/dentin, PDL and cementum in the root portion **(M)**. Haematoxylin eosin staining of PDL and alveolar bone in the crown **(L)** and root portions **(N)**. Expression of biglycan in dentin **(K1)**, PDL **(K1,L1**) and alveolar bone **(L1)** in the crown portion; predentin/dentin/cementum **(M1)**, PDL **(M1,N1)** and alveolar bone **(N1)** in the root portion. Expression of collagen XIIa in dentin **(K2)**, PDL **(K2,L2)** and alveolar bone **(L2)** in the crown portion; predentin/dentin/cementum **(M2)**, PDL **(M2,N2)** and alveolar bone **(N2)** in the root portion. Expression of collagen XIVa in dentin **(K3)**, PDL **(K3,L3)** and alveolar bone **(L3)** in the crown portion; predentin/dentin/cementum **(M3)**, PDL **(M3,N3)** and alveolar bone **(N3)** in the root portion. Expression of decorin in dentin **(K4)**, PDL **(K4, L4)** and alveolar bone **(L4)** in the crown portion; predentin/dentin/cementum **(M4)**, PDL **(M4,N4)** and alveolar bone **(N4)** in the root portion. Expression of fibromodulin in dentin **(K5)**, PDL **(K5,L5)**, and alveolar bone **(L5)** in the crown portion; predentin/dentin/cementum **(M5)**, PDL **(M5,N5)**, and alveolar bone **(N5)** in the root portion. Pd, Predentin; d, dentin; pdl, periodontal ligaments; ab, alveolar bone; ce, cementum; BGN, biglycan; COL12, collagen type XIIa; COL14, collagen type XIVa; DCN, decorin; FMOD; fibromodulin; HE, haematoxylin/eosin. Scale bar = 25 μm.

**Figure 6:**
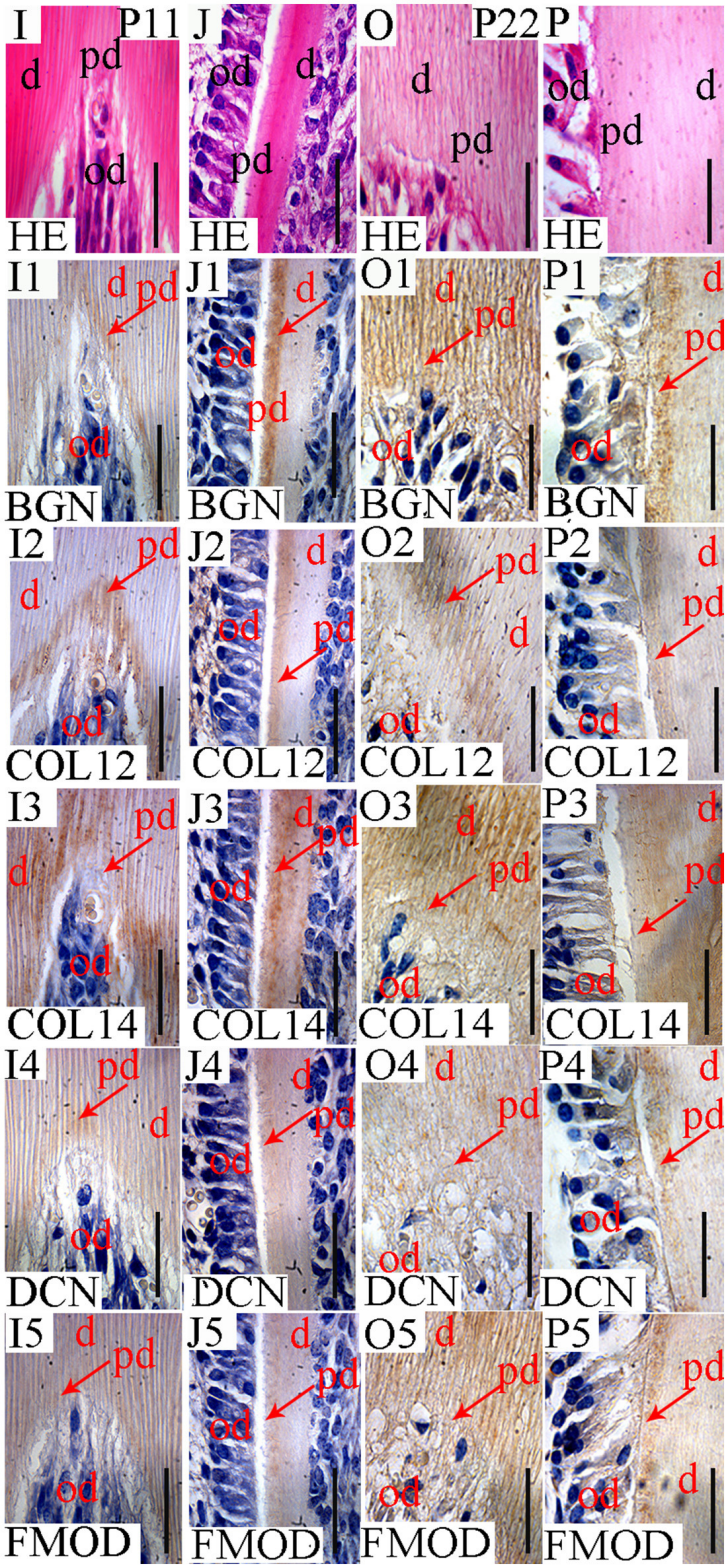
Localization of FACITs and SLRPs in predentin and dentin at P11 and P22. Detail of dentin and predentin in crown **(I)** and root portions **(J)** at P11. Detail of dentin and predentin in the crown **(O)** and root portions **(P)** at P22. Detection of biglycan in predentin, dentin in the crown **(I1)** and root portions **(J1)** at P11. Presence of collagen XIIa in predentin in the crown **(I2)** and root portions **(J2)** at P11. Detection of collagen XIVa in predentin in the crown **(I3)** and root portions **(J3)** at P11. Expression of decorin in predentin/dentin in the crown **(I4)** and root portions **(J4)** at P11. Presence of fibromodulin in predentin/dentin in the crown **(I5)** and root portions **(J5)** at P11. Detection of biglycan in predentin/dentin in the crown **(O1)** and root portions **(P1)** at P22. Expression of collagen XIIa in predentin in the crown **(O2)** and root portions **(P2)** at P22. Detection of collagen XIVa in predentin/dentin in the crown **(O3)** and root portions **(P3)** at P22. Presence of decorin in predentin/dentin in the crown portion **(O4)** and predentin in the root portion **(P4)** at P22. Localization of fibromodulin in predentin/dentin in the crown portion **(O5)** and root predentin **(P5)** at P22. D, Dentin; pd, predentin; od, odontoblast; BGN, biglycan; COL12, collagen type XIIa; COL14, collagen type XIVa; DCN, decorin; FMOD; fibromodulin; HE, haematoxylin/eosin. Scale bar = 25 μm.

**Figure 7:**
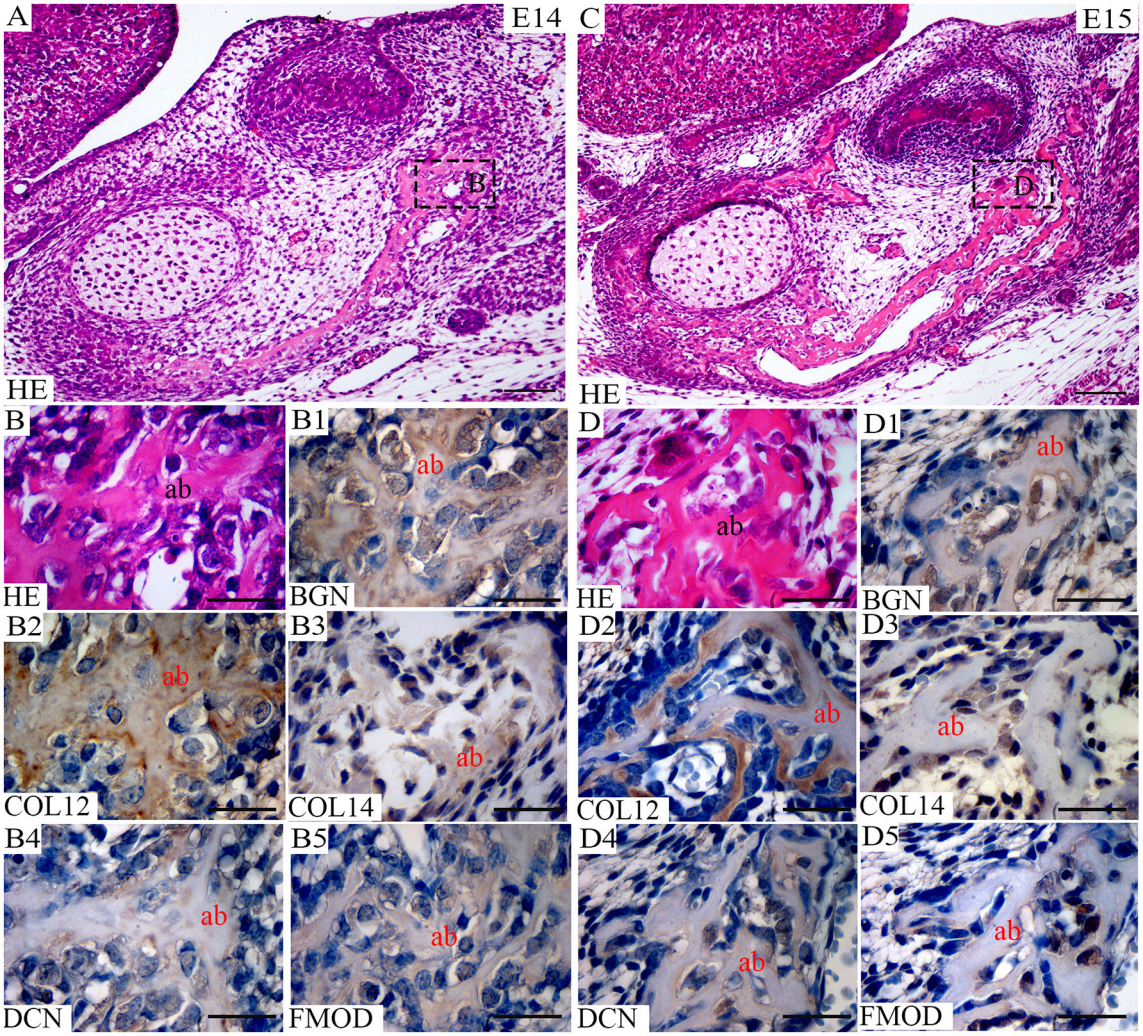
Co-expression of FACITs and SLRPs in early alveolar bone development. **(A)** Haematoxylin and eosin staining of embryonic tooth and surrounding tissues at E14. **(B)** Detail of alveolar bone at E14. **(C)** Haematoxylin and eosin staining of embryonic tooth and surrounding tissues at E15. **(D)** Detail of alveolar bone at E15. Expression of biglycan **(B1)**, collagen XIIa **(B2)**, collagen XIVa **(B3)**, decorin **(B4)**, fibromodulin **(B5)** in developing alveolar bone at E14. Expression of biglycan **(D1)**, collagen XIIa **(D2)**, collagen XIVa **(D3)**, decorin **(D4)**, fibromodulin **(D5)** in developing alveolar bone at E15. Ab, Alveolar bone; BGN, biglycan; COL12, collagen type XIIa; COL14, collagen type XIVa; DCN, decorin; FMOD; fibromodulin; HE, haematoxylin/eosin. Scale bar = 25 μm.

In this study, the localization of five antigens involved in collagen fibrillogenesis was investigated at selected stages, from the bud stage of molar tooth development until eruption with focus on specific structures: (1) soft tissues including dental pulp and periodontal ligament, (2) hard tissues including alveolar bone and dentin and (3) junctions between periodontal ligament and alveolar bone or cementum. At E18 and P0 (Figures 1, 2), the first molar had reached the bell stage and was encapsulated in the alveolar bone, odontoblasts became functional, secreting predentin/dentin. The study was extended to P11, before tooth eruption, and P22, after eruption (Figure 3). At P11, the tooth crown was covered by predentin and dentin, which also extended in the root portion. The periodontal ligament was formed and its cells were in contact with root dentin on one side and alveolar bone on the other side (Figure 4, details in Figure 6). At P22, roots had elongated and cells of the periodontal ligament were in contact with acellular cementum in the coronal portion of the root while with cellular cementum in its apical part. At their other extremity, periodontal ligament fibers interacted with alveolar bone (Figure 5, details in Figure 6). Stages E14 and E15 (Figure 7) were also investigated to determine the initial patterning of the five antigens at the onset of the alveolar bone formation.

### Temporospatial localizations of collagens XIIa and XIVa along with biglycan, decorin and fibromodulin in soft tissues: the dental pulp and periodontal ligament

No staining was detected in dental tissues at E14-15, neither for collagen XIIa (data not shown), nor for collagen XIVa (data not shown). At E18, collagen XIIa and XIVa were localized in the ECM embedding dental pulp cells (Figures 1B, 2D). At the cusp tip, the two FACITs were also expressed by differentiated odontoblasts (Figure 2D). BGN, DCN, and FMOD co-distributed with collagens XIIa and XIVa (Figures 1B, 2D).

Collagens XIIa and XIVa were also present in the peridental mesenchyme at E18 (Figure 1C). The intensity of the signals was about the same in the dental (Figure 2D) and peridental mesenchymes (Figure 1C). In the peridental mesenchyme, the signal was positive for FMOD and almost negative for BGN and for DCN (Figure 1C). At P0, the staining for collagen XIIa in the dental pulp ECM became much stronger than the one for collagen XIVa (Figure 1F).

This difference was maintained at P11 (Figure 3B) and P22 (Figure 3D). Such a developmentally regulated change in the balance between the two FACITs after E18 in the dental pulp was not apparent when comparing the stainings for the 3 different SLRPs (Figures 1F, 3B,D).

In the PDL, the presence of collagen XIIa was detected already at P11 (Figures 4E–H), thus before tooth eruption. At that stage, the signal for collagen XIVa was much weaker (Figures 4E–H). At P22, when the organization of PDL cells and fibers had progressed, the intensity of the stainings for the two FACITs was very similar (Figures 5K–N). In the PDL at P11, BGN, and DCN were almost negative, while FMOD showed a week staining (Figures 4E–H). The staining for all three antigens became much stronger at P22 (Figures 5K–N).

### Temporospatial localizations of collagens XIIa and XIVa along with biglycan, decorin and fibromodulin in hard tissues: the alveolar bone and predentin/dentin

At E18, the staining for collagen XIIa in the mesenchymal cells in between the alveolar bone trabeculae was more intense than for collagen XIVa (Figure 1C). However, in both cases, the signals had decreased compared to what was observed at E15 (Figures 1C, 7D). At E18, the stainings for BGN, DCN, and FMOD in alveolar bone matrix was much stronger than for collagens XIIa and XIVa (Figure 1C). The five antigens were present in predentin at E18 (Figure 2D). They were maintained in the crown at P0 (Figure 2H), as in the root portion (Figure 1F). At P0, collagen XIIa, BGN, and DCN (Figures 1F, 2H) were mostly restricted to the predentin area, while collagen XIVa and FMOD extended further toward dentin (Figure 2H). The five antigens maintained the same patterns at P11, in the crown (Figure 6I) as in the root dental matrix (Figure 6J). Except for collagen XIIa in mature alveolar bone matrix, all other antigens were detected in the alveolar bone matrix at P11 (Figures 4F,H). There, very weak stainings was observed for BGN and DCN, while much stronger for collagen XIVa and FMOD (Figures 4F,H). When comparing the stainings for collagens XIIa and XIVa in the alveolar bone matrix, the signal for collagen XIIa was stronger at E18 (Figure 1C), while collagen XIVa became prominent at P0 (Figure 1G). BGN, DCN, and FMOD were observed in alveolar bone matrix at this stage (Figure 1G). Stainings in predentin did not show the same variations.

At P22, collagens XIIa as well as XIVa, are constituents of alveolar bone matrix (Figures 5L,N). As observed in predentin, the staining for collagen XIVa in alveolar bone was stronger than for collagen XIIa (Figures 5L,N). The progressive increase of staining for collagen XIIa compared to XIVa, as observed in the dental pulp (Figure 3D), PDL (Figures 5K–N), predentin (Figures 3D, 5M, 6O,P), and was not visible in bone matrix (Figures 5L,N). BGN, DCN, and FMOD (Figures 5L,N) were also present in the alveolar bone matrix at P22. They showed differences in the intensity of labeling. DCN and FMOD showed a weaker signal than BGN (Figures 5L,N). At P22, a strong signal for BGN was also detected in crown and root predentin, where DCN and FMOD showed a weaker signal. All three antigens were absent from dentin matrix, both in the crown and root portions (Figures 6O,P).

Alveolar bone formation becomes morphologically apparent at E13. At this stage, none of the five antigens could be detected. The positive staining of bone matrix for collagen XIIa was observed at E14 (Figure 7B) and was maintened also at E15 (Figure 7D). Collagen type XIIa persisted in the alveolar/mandibular bone throughout development. Together with collagen XIIa, collagen type XIVa protein was detected in the early alveolar bone at E14 (Figure 7B) and E15 (Figure 7D). All investigated SLRPs were already present in the alveolar bone matrix at E14 (Figure 7B) and maintained also at E15 (Figure 7D). At these two stages, the alveolar bone matrix was heterogeneously labeled.

### Temporospatial localizations of collagens XIIa and XIVa along with biglycan, decorin and fibromodulin between non-mineralized and mineralized tissues: interactions between periodontal ligament and alveolar bone or cementum

At P11, collagen XIIa was expressed by cells in central part of the PDL or in contact with the alveolar bone (Figures 4E–H). Much less collagen XII was expressed by PDL cells in contact with the root matrix (Figure 4G). At this stage, the staining for collagen XIVa in the PDL itself was very weak (Figures 4E–H). FMOD also showed an asymmetrical patterning, similar to that of collagen XIIa (Figures 4E,F). A weak but homogeneous signal was detected for DCN and BGN (Figures 4E–H) within PDL cells. Collagen XII was still present in the PDL at P22 (Figures 5K–N), no longer showing the asymmetrical patterning as observed at P11. When moving along the root at P22, the staining for collagen XIIa showed variations at the PDL cementum/dentin junction (Figures 5K,M). Differences were also observed with collagen XIVa, including a positive line precisely at the interface between PDL cells and the root matrix, visible in the coronal part (Figure 5K), but not in the apical part of the root (Figure 5M).

At P22, the cellular cementum was negative for collagen XIIa, but positive for collagen XIVa (Figure 5M). Collagen XIIa was expressed by PDL cells in contact with the alveolar bone (Figure 5N) as well as by PDL cells in contact with the acellular cementum (Figure 5K). At P22, BGN, DCN, and FMOD (Figure 5M) were detected in the cellular cementum.

## Discussion

Several fibril-forming collagens are present in the dental pulp, predentin, and dentin, as well as in the PDL and alveolar bone. Collagen fibrillogenesis can occur spontaneously, but *in vivo* it needs to be regulated by FACITs and SLRPs (Chen and Birk, 2013; for review, see Kalamajski and Oldberg, 2010; Ricard-Blum, 2011).

The patterning and timing of expression of five molecules involved in the regulation of collagen fibrillogenesis will be discussed according to observations made in two distinct soft tissues, in two different mineralizing matrices (bone and predentin/dentin), and in the PDL comparing its junction with cementum or with alveolar bone.

### Timing of expression and localization of collagens XIIa and XIVa in the ECM of unmineralized tissues: dental pulp vs. periodontal ligament

Collagen XIIa, together with the three SLRPs, as detected here in the dental pulp at this stage, might be involved in the regulation of collagen fibrillogenesis by modulating fibril packing (Chen et al., 2015). All three SLRPs investigated here bind collagens XIIa and XIVa via either their protein core or proteoglycan moieties (Font et al., 1993, 1998; Ehnis et al., 1997). Since the five antigens were detected in the dental pulp, the three SLRPs could interfere with collagens XIIa and XIVa to modulate collagen I fibrillogenesis. Although, possibly having different binding sites on collagen fibrils, functional redundancies have been demonstrated between different SLRPs (Chen and Birk, 2013). The protein cores of SLRPs are critical to regulate collagen fibrillogenesis (Rada et al., 1993) and lateral growth of the fibrils (Nurminskaya and Birk, 1998), whereas glycosaminoglycans can bridge neighbor fibrils (Henninger et al., 2007; Lewis et al., 2010).

According to a study performed by ISH, during mouse odontogenesis, transcripts for collagen XIIa were first detected from P8 in PDL cells located next to the alveolar bone (MacNeil et al., 1998). At that stage, no signal was detected in dental tissues (MacNeil et al., 1998). Immuostainings as observed here gave very different results. Collagens XIIa and XIVa were already detected in the peridental mesenchyme at E18, and the same intensity was observed for the two FACITs. It thus differed from immunostaining for collagen type I, which was much stronger in the peridental mesenchyme compared to the dental papilla (Lesot et al., 1981).

Collagen type I is the most abundant fibrillar collagen in the PDL. Collagen type I fibrils play a critical role in establishing the mechanical properties of the PDL (Kaku and Yamauchi, 2014). The presence of both collagens XIIa and XIVa in the PDL raises the question of their respective roles there. MacNeil et al. (1998) showed that collagen XIIa was first expressed in the dental follicle/PDL region during tooth eruption and then increased in the PDL as the molar tooth erupted and achieved occlusal contact. In fact, collagen XIIa was detected in the PDL already at P11, thus before tooth eruption. These observations suggested that the expression of collagen XIIa coincided with the alignment and organization of PDL fibers.

Our immunostainings showed progressively increased expression of FMOD and DCN in the PDL, from P11 to P22. These observations are in agreement with previous data suggesting their interaction with collagen XII (Matias et al., 2003; Matheson et al., 2005; Leong et al., 2012; Wang et al., 2014). Other SLRPs, including lumican and asporin, have also been detected in the PDL (Nakamura et al., 2005; Leong et al., 2012; Wang et al., 2014). Matheson et al. (2005) analyzed the periodontal tissues of mice harboring targeted deletions of DCN, FMOD, or lumican genes and also in lumican and FMOD double knockout mice. Histology and ultrastructural analyses showed that all these gene deletions resulted in a unique fibril and fibril bundle phenotype, although tooth eruption was not impaired (Matheson et al., 2005). Abnormal collagen fibrils have also been observed in the PDL of mice with double deficiency for FMOD and BGN (Wang et al., 2014). DCN has been suggested not only to regulate the organization of collagen fibrils but also to be involved in PDL homeostasis by regulating cell proliferation (Häkkinen et al., 2000; Wang et al., 2014). In the adult, all these antigens are still present in the PDL (Salmon et al., 2016). Asporin is considered a negative regulator of mineralization, specific for the PDL (Leong et al., 2012).

### Situation in the alveolar bone and comparison with predentin/dentin

It is not clear why collagens XIIa and XIVa come together during alveolar bone formation, except if they have different functions or molecular associations, as suggested previously in different contexts (Shaw and Olsen, 1991; Young et al., 2000). Investigations of long bone formation in Col12a1 −/− mice and in cultured osteoblasts have led to the conclusion that collagen XII is necessary for proper osteoblast/osteocyte differentiation (Izu et al., 2011; Chiquet et al., 2014). Apparently, the situation is different in the alveolar bone, since it was detected early after bone formation was initiated. The hypothesis that collagen XIIa might be involved in bone mineralization has also been proposed, but not demonstrated yet (Alves et al., 2013). Genes encoding for DCN and BGN are expressed by osteoblasts at early stages (E16) of mandibular bone formation in rats (Kamiya et al., 2001). According to Kamiya et al. (2001), the strongest signal was observed for DCN in the osteoid, and both DCN and BGN proteoglycans were expressed by osteoblasts before mineralization started.

While already detected in predentin at E18, all five antigens showed distinct change in their patterning at P0. At that stage when dentin was apparent, collagen XIIa, BGN, and DCN were mostly restricted to the predentin area, whereas collagen XIVa and FMOD extended further toward the dentin. FMOD has been reported to be highly expressed in mineralizing tissues (Young et al., 2002).

The patterning of the two FACITs and three SLRPs was different in bone, mainly when mineralized. Comparing the ultrastructure of different tissues from bgn- and dcn-deficient mice, Corsi et al. (2002) showed that the effects on collagen fibril structure were similar in the dermis but not in bone. These authors also showed the existence of synergies between the two SLRPs in determining collagen fiber size in the bone (Corsi et al., 2002).

The different variations in the stainings in bone matrix, when compared to predentin, illustrated developmentally regulated changes in the balance of the two FACITS. These changes were also tissue-specific. Other examples, where the expression patterns of FACITs were obviously developmentally regulated, have suggested their functions are not to be restricted only to structural ones (Ricard-Blum, 2011). Mice with double deficiency of FMOD and BGN exhibited an altered remodeling of the alveolar bone, together with an elevated number of osteoclasts (Wang et al., 2014). These authors suggest two non-exclusive mechanisms to explain their observations: (a) direct interactions between SLRPs and fibrillar collagen are known to protect it from degradation by MMPs (Geng et al., 2006), and (b) alteration of TGFβ/BMP signaling, which SLRPs can modulate (Schaefer and Schaefer, 2010).

### Junctions between non-mineralized and mineralized tissues: tooth attachment

Fibrillar collagens, mainly collagen type I, are expressed by PDL cells in contact with two distinct mineralized matrices: the cementum and alveolar bone. Collagen fibers (Sharpey's fibers) in the PDL are continuous from their insertion sites in the alveolar bone at one extremity to their insertion into the cementum–root dentin junction at the other end. Proteoglycans are present all along (Ho et al., 2007). At their insertion sites into either the alveolar bone or cementum, the PDL fibers are mineralized (McKee et al., 2013).

FACITs as well as SLRPs are expressed by PDL cells in contact with either the alveolar bone or the cementum. The asymmetric patterning of collagen XIIa might simply reflect the fact that although alveolar bone had started to form 2 weeks earlier at E14, cementogenesis was only in progress at P11. FMOD, but not DCN, also showed an asymmetrical patterning, similar to that of collagen XIIa. However, this occurred at the apical part of the root, suggesting a relationship with cementogenesis. SLRPs should play complementary roles, since defects of collagen fibrillar and suprafibrillar organization in the PDL have been observed in mice with targeted deletions in DCN and FMOD genes (Matheson et al., 2005). Collagen fibers from the PDL anchor to the acellular cementum, thus mediating tooth attachment to the surrounding alveolar bone. The insertion of PDL collagen fibrils in the cementum was clearly visible using cryo-TEM (Quan and Sone, 2015). The expression of collagen XIIa in the PDL from rats has been reported to increase from P25 (when root formation is not achieved) to P40 (when teeth are functional; Karimbux et al., 1992). This suggested that collagen XIIa might be involved in the 3D organization of the ECM in the PDL. This hypothesis was later supported by analyzing the PDL matrix organization in transgenic young adult mice carrying a mutation in the gene encoding for collagen XII (Reichenberger et al., 2000). SLRPs interacting with collagen XIIa, such as FMOD and DCN, are also present in the PDL (Matias et al., 2003; Matheson et al., 2005; Leong et al., 2012; Wang et al., 2014). At P22, BGN, DCN, and FMOD were detected in the cellular cementum, which differs from the observations published by Matheson et al. (2005). These authors found the cellular cementum negative for DCN and almost negative for BGN and FMOD.

In conclusion, the dental mesenchyme and mesenchymal cells from the PDL show major differences. Indeed, the first odontoblasts differentiate and become postmitotic 1 day before birth in the mouse first lower molar. They will survive all life long if no carious lesions develop (Smith and Lesot, 2001), while cells from the PDL show a high turnover (Trombetta and Bradshaw, 2010). Cells and collagen fibers in the PDL are submitted to strong mechanical stresses compared to dental pulp cells in general, which are all surrounded and thus protected by mineralized matrices. Surprisingly then, the expression and patterns of the regulators of collagen fibrillogenesis did not show major changes when comparing the two soft tissues. The asymmetrical patterning of collagens XIIa and XIVa, as observed in PDL cells in contact with mineralized matrices, might be correlated with the different timings when comparing the formation of alveolar/mandibular bone, which starts at E14, and cementogenesis, which is initiated postnatally. The contact between peridental mesenchymal cells and the forming alveolar bone thus exists several days before the PDL starts to form and organizes histologically. For all tissues considered in this study, SLRPs showed a more complex patterning, and their variation in space or intensity during development was distinct from that observed for collagens XIIa and XIVa. The reason might be that, besides their involvement in regulating collagen fibrilogenesis, SLRPs also intervene as extracellular binders and modulators for several signaling molecules (Schaefer and Iozzo, 2008; Wang et al., 2014).

## Author contributions

IZ student, experimental work (immunohistochemistry, correlation analysis), contribution to ms preparation. EM project idea, experimental design, supervision of experimental work, contribution to ms finalization. HL development of project idea, theoretical background, ms preparation.

### Conflict of interest statement

The authors declare that the research was conducted in the absence of any commercial or financial relationships that could be construed as a potential conflict of interest.

## Footnotes

^1^Available at: http://www.ncbi.nlm.nih.gov/gene/ [Accessed May 25, 2016].

## Supplementary material

Supplementary Figure 1 Negative controls of the immunohistochemical staining. **(A)** Dental pulp and predentin at P0. Immunohistochemical detection of biglycan **(A1)**, collagen type XII **(A2)**, collagen type XIV **(A3)**, decorin **(A4)**, fibromodulin **(A5)**, and the negative control **(A6)**. **(B)** Periodontal ligament and alveolar bone at P0. Immunohistochemical detection of biglycan **(B1)**, collagen type XII **(B2)**, collagen type XIV **(B3)**, decorin **(B4)**, fibromodulin **(B5)**, and the negative control **(B6)**. **(C)** Dentin, predentin and odontoblasts at P0. Immunohistochemical detection of biglycan **(C1)**, collagen type XII **(C2)**, collagen type XIV **(C3)**, decorin **(C4)**, fibromodulin **(C5)**, and the negative control **(C6)**.
