# Supplementary figures and images for: Regulators of Collagen Fibrillogenesis during Molar Development in the Mouse
Source: Front Physiol. 2017 Aug 2;8:554. doi: 10.3389/fphys.2017.00554 (PMC5539247; doi:10.3389/fphys.2017.00554)

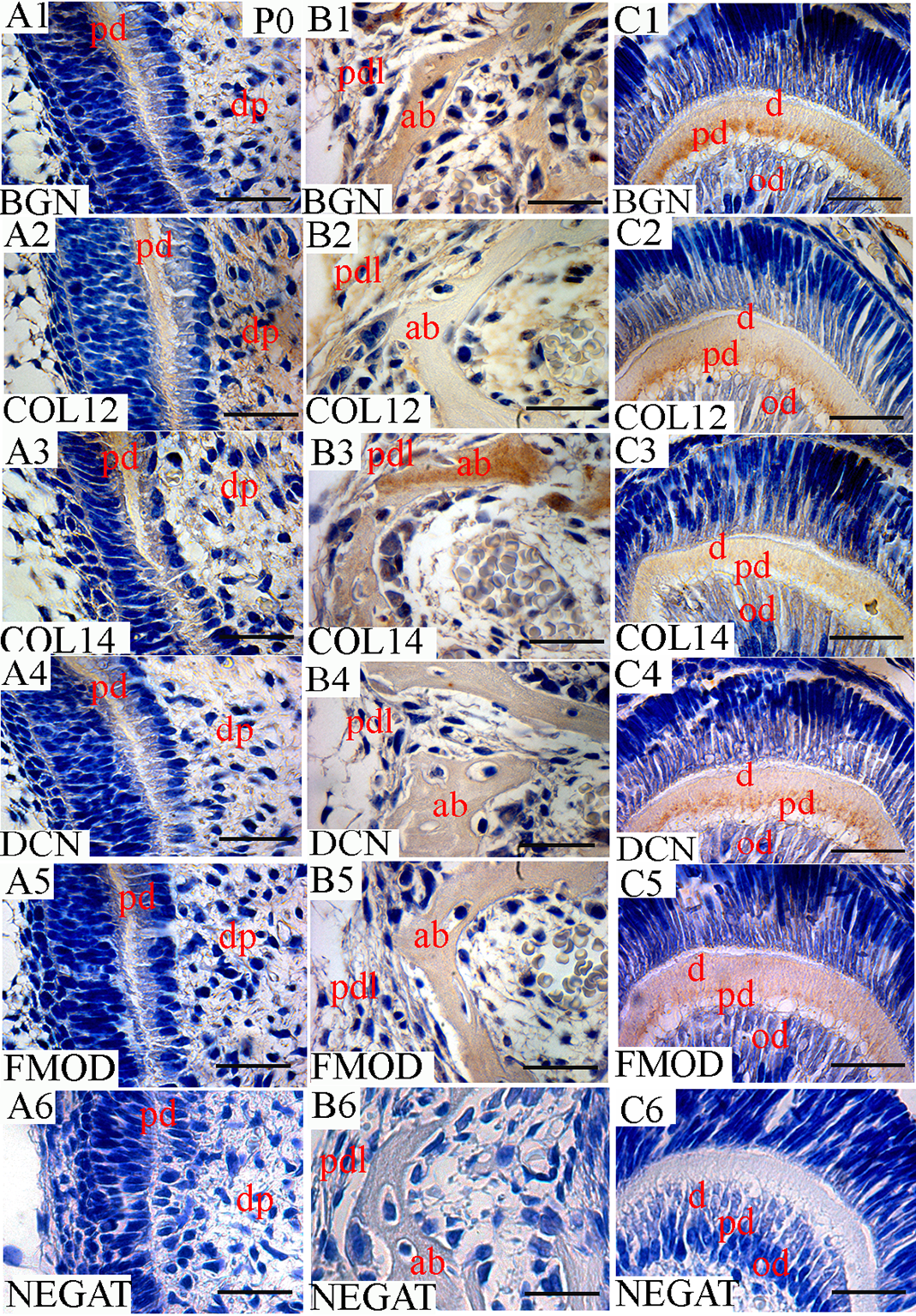

Supplement: Supplementary Figure 1 — Negative controls of the immunohistochemical staining. (A) Dental pulp and predentin at P0. Immunohistochemical detection of biglycan (A1), collagen type XII (A2), collagen type XIV (A3), decorin (A4), fibromodulin (A5), and the negative control (A6). (B) Periodontal ligament and alveolar bone at P0. Immunohistochemical detection of biglycan (B1), collagen type XII (B2), collagen type XIV (B3), decorin (B4), fibromodulin (B5), and the negative control (B6). (C) Dentin, predentin and odontoblasts at P0. Immunohistochemical detection of biglycan (C1), collagen type XII (C2), collagen type XIV (C3), decorin (C4), fibromodulin (C5), and the negative control (C6). [file Image1.TIF]
